# Development of a Bloodstream Infection Surveillance Programme at a Resource-Limited South African Neonatal Unit

**DOI:** 10.3390/antibiotics14040392

**Published:** 2025-04-10

**Authors:** Frances Ashton, Adrie Bekker, Magdalena Aucamp, Kessendri Reddy, Andrew Whitelaw, Angela Dramowski

**Affiliations:** 1 Department of Paediatrics and Child Health, Faculty of Medicine and Health Sciences, Stellenbosch University, Cape Town 7500, South Africa; 2 Unit for Infection Prevention and Control, Tygerberg Hospital, Cape Town 7500, South Africa; magdalena.aucamp@westerncape.gov.za; 3 Division of Medical Microbiology and Immunology, Department of Pathology, Faculty of Medicine and Health Sciences, Stellenbosch University, Cape Town 7500, South Africa

**Keywords:** neonate, bloodstream infections, antimicrobial resistance, empiric antibiotic, sepsis

## Abstract

**Background**: Data from African neonatal units conducting bloodstream infection (BSI) surveillance is limited. **Methods**: Prospective clinical and laboratory surveillance of incident BSI episodes was conducted among in-patients at the 132-bed neonatal service at Tygerberg Hospital, Cape Town, South Africa (2017–2021), describing patient demographics, BSI rates, pathogen profiles, and empiric antibiotic concordance rates. **Results:** In total, 842 BSI episodes were identified in 740 neonates; most were preterm (661/740; 89.3%) and of low birth weight (640/740; 86.5%). The early onset BSI rate (<3 days of life) was 2.9/1000 live births, with *S. agalactiae*, *K. pneumoniae*, and *E. coli* predominating. Over time, ampicillin plus gentamicin coverage rates for early onset BSI pathogens declined from 93.8% to 63.6%. The healthcare-associated BSI rate (onset >3 days of life) was 3.4/1000 in-patient days, with *K. pneumoniae*, *S. aureus*, and *S. marcescens* predominating. Antibiotic coverage rates for healthcare-associated BSIs improved over time, from 72.2% to 89.2% (piperacillin plus amikacin) and from 68.1% to 84.6% (meropenem). Nearly one-third of BSI episodes were fatal (244/842; 29.0%), with two-thirds of these deaths considered BSI-attributable. Gram-negative BSIs increased mortality (OR 2.88; 95% CI 1.93–4.32) compared to Gram-positive BSIs (*p* < 0.001). Discordant empiric antibiotic therapy (OR 1.55; 95% CI 1.10–2.17) increased the risk of death compared to concordant therapy (*p* = 0.012). **Conclusions**: Neonatal BSI surveillance demonstrated that Gram-negative pathogens remain important causes of early onset and healthcare-associated BSIs in this resource-limited neonatal service. Declining coverage rates for empiric antibiotics prescribed for early onset BSI highlight the need for a change in treatment guidelines to minimise discordant therapy.

## 1. Introduction

More than 90% of neonatal infection-related deaths occurred in low-and middle-income countries (LMIC) in 2019 [1]. The burden of neonatal infections is highest in sub-Saharan Africa [2], where the overall neonatal mortality rate of 27 per 1000 live births still far exceeds the Sustainable Development Goal of <12 per 1000 live births [3]. Apart from causing mortality, neonatal infections also increase the need for intensive care, prolong hospital stays, increase healthcare costs, and cause long-term neurodevelopmental disability in survivors [4,5].

Bacterial infections in neonates are typically classified as either early onset neonatal sepsis (EONS—infections occurring within 72 h of birth) or healthcare-associated infection (HAI—occurring after 72 h of life) [6]. In several high-income countries (HICs), surveillance systems are used to collect neonatal infection data systematically and continuously, usually focusing on the most prevalent neonatal HAI type, bloodstream infections (BSIs) [7,8,9,10]. The epidemiology of neonatal infections varies by region, facility type, and population, and therefore data and practices from HICs cannot be generalised to LMIC neonatal units [10].

There is currently no standardised neonatal infection surveillance system in South Africa or any other African country, although several hospitals have published data on neonatal unit BSI epidemiology [5,11,12,13,14]. The first national South African retrospective laboratory neonatal BSI analysis (2014–2019) showed 82% of neonatal BSI episodes to be HAI, with Gram-negative organisms such as *Klebsiella pneumoniae* and *Acinetobacter baumannii* predominating [15]. Declining susceptibility to empiric antibiotic regimens for EONS (ampicillin/penicillin G plus gentamicin or a third-generation cephalosporin) and HAI (piperacillin-tazobactam plus amikacin [11] or meropenem) has also been documented [15]. Regularly updated institution- or region-specific data on antimicrobial resistance (AMR) in BSI pathogens is crucial to ensure that empiric antibiotic regimens provide appropriate coverage [15,16]. BSI surveillance data is also extremely useful in detecting and tracking neonatal infection outbreaks, as described in reports from several national and institutional outbreaks in South Africa [13,17].

Given the clinical usefulness of neonatal BSI surveillance data and the paucity of published data from Africa, we describe the development, implementation, and findings of a prospective neonatal BSI surveillance programme at a South African public hospital neonatal unit.

## 2. Methods

### 2.1. Study Design and Population

Tygerberg Hospital (TBH) is a central academic hospital in Cape Town, South Africa. The neonatal service has 132 beds, including 12 neonatal intensive care unit (NICU) beds, three general neonatal wards, and a kangaroo mother care ward, providing medical and surgical services to approximately 5000 neonates annually [18]. The hospital’s obstetric unit provides care for high-risk pregnancies and delivers almost 8000 live births per year, with a 42% low-birth-weight rate [18]. The hospital has a dedicated unit for infection prevention and control (IPC), including an IPC nurse practitioner assigned to the neonatal, paediatric, and obstetric wards to conduct IPC training, audits, surveillance, and outbreak management. Previously, retrospective analysis of laboratory-based surveillance data from TBH (2009–2018) identified a high burden of Gram-negative infections and a decline in the in vitro activity of piperacillin-tazobactam plus amikacin [11]. In 2017, the TBH Unit for IPC developed and implemented a neonatal BSI surveillance programme, with support from the neonatology, paediatric infectious disease, and microbiology divisions.

### 2.2. BSI Episode Identification and Clinical Data Collection

The surveillance method, programme implementation, and findings from the first five years of the prospective neonatal BSI surveillance programme (1 January 2017–31 December 2021) are reported. The BSI surveillance dataset was compiled in real time by the neonatal unit IPC practitioner, using automated electronic BSI line lists received daily via email from the National Health Laboratory Service’s (NHLS) central data warehouse. The eligibility criteria for inclusion in the neonatal BSI surveillance programme were (1) any inborn or outborn neonate (<10 days of life at the time of hospital admission), (2) admitted to the NICU or any neonatal ward, (3) with a blood culture yielding a recognised pathogen/s. Some infants with a prolonged hospital stay (e.g., preterm infants) remained eligible for inclusion even if BSI onset occurred >27 days of life, as long as the infant was still admitted to the neonatal service. Duplicate positive blood cultures within 14 days of the original isolate were removed, and blood culture contaminants were excluded [19]. BSIs with more than one pathogen cultured on the same specimen were classified as polymicrobial infections but counted as a single BSI episode. The IPC nurse practitioner verified the emailed BSI reports with the microbiology laboratory, to confirm pathogen identity, AMR profile, and patient location. For all BSI episodes, the IPC nurse obtained clinical information on patient demographics, empiric antimicrobial treatments, and outcomes. Final antimicrobial susceptibility data was obtained retrospectively from the LabTRAK NHLS data server.

### 2.3. Study Definitions

Patients were classified by gestational age at birth: term (≥37 weeks), moderate–late preterm (32–36 6/7 weeks), very preterm (28–31 6/weeks), and extremely preterm (<28 weeks). A diagnosis of a BSI episode was made for positive blood cultures yielding a known neonatal pathogen/s identified using standard paediatric BacT/Alert bottles and Vitek identification and antimicrobial susceptibility testing. BSI pathogens and contaminants were classified using the National Healthcare Safety Network list [19], and samples that cultured contaminants were not included in the dataset. Coagulase-negative staphylococcus aureus was included as a pathogen if the same species was isolated from 2 separate blood cultures taken at least 24 h apart. Early onset BSI (EO-BSI) was defined as a BSI diagnosed from a culture collected before 72 h of life. The EO-BSI rate was calculated as the total EO-BSI episodes as a proportion of the total live births times 1000. Healthcare-associated BSI (HA-BSI) was defined as a BSI occurring after 72 h of life, and the rate was calculated as the number of HA-BSIs per neonatal in-patient days times 1000. BSI attributable mortality was defined as death occurring within 3 days of blood culture collection [20]. The hospital’s empiric antibiotic regimen for EO-BSI is ampicillin/penicillin G plus gentamicin or a third-generation cephalosporin, with the use of piperacillin-tazobactam plus amikacin for HA-BSI in stable neonates and meropenem in critically ill neonates/suspected healthcare-associated meningitis. During the carbapenem-resistant *Acinetobacter baumannii* (CRAB) outbreak in 2017 and carbapenem-resistant Enterobacterales (CRE) outbreak in 2019, selected cases were commenced empirically on colistin-containing regimens in discussion with the paediatric infectious disease team. Empiric antibiotic therapy was defined as the initial antibiotic/s commenced at the time of blood culture sampling. An antibiotic regimen was defined as concordant if the pathogen was susceptible to at least one antimicrobial in the empiric regimen, whereas a discordant regimen was defined when a pathogen was not susceptible to any antibiotic in the empiric regimen, i.e., bug–drug mismatch.

### 2.4. Study Approvals, Data Management, and Statistical Analysis

Research approvals were obtained from the Stellenbosch University Health Research Ethics Committee and hospital management (HREC S22/04/063), with a waiver of individual informed consent. The BSI surveillance dataset was obtained from the Unit for IPC, as a password-protected Excel spreadsheet, and verified using hospital information systems, including electronic discharge records and scanned repositories of the medical records. Prior to analysis, the dataset was anonymised using randomly generated study numbers. The demographic characteristics were summarised using standard descriptive summaries. Categorical variables such as sex were reported as frequencies and percentages, while numeric variables such as age in days were summarised using means or medians depending on their distribution. The Chi–squared test was used to compare categorical data. The proportion of BSI episodes receiving concordant empiric antimicrobial therapy was calculated for each year (2017–2021). For all comparisons, a *p*-value of 0.05 was considered significant. Data was analysed using STATA IC v17.

## 3. Results

### 3.1. Bloodstream Infection Classification and Incidence Density

**Figure 1:**
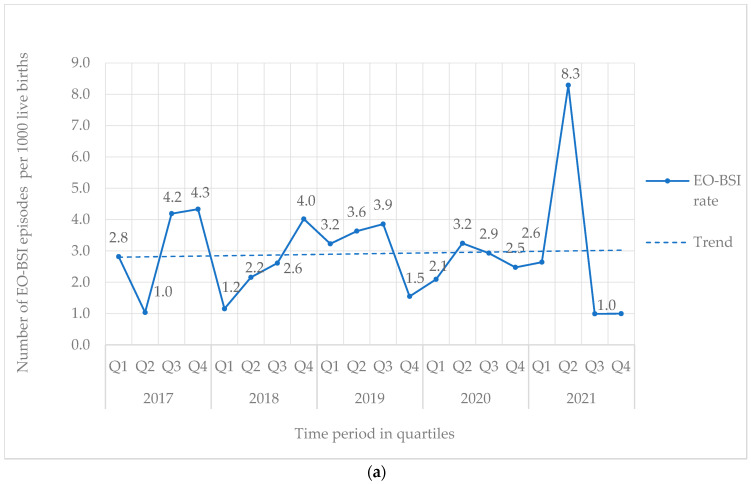

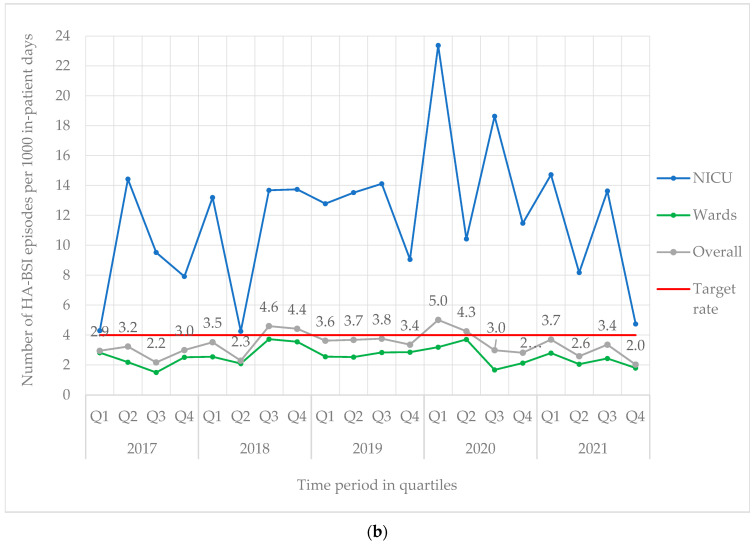
(**a**) Quarterly EO-BSI rate per 1000 live births (2017–2021). EO-BSI, early onset bloodstream infection. (**b**) Quarterly HA-BSI rate per 1000 in-patient days, stratified by neonatal unit location (2017–2021). EO-BSI, early-onset bloodstream infection. HA-BSI, healthcare-associated bloodstream infection; NICU, neonatal intensive care unit.

From 2017 to 2021, 842 BSI episodes were identified in 740 neonates (Table 1). EO-BSI accounted for 13.4% (113/842) of BSI episodes, with an incidence density of 2.9 per 1000 live births. A substantial elevation in the incidence density of EO-BSIs was detected in 2021 (quarter two) to 8.3 per 1000 live births with clusters of *Streptococcus agalactiae* and *K. pneumoniae* infections (Figure 1a). Overall, most BSI episodes were HA-BSI (86.6%; 729/842), with an incidence density of 3.4 per 1000 in-patient days. The HA-BSI rate in the NICU was up to seven-fold higher compared to the general neonatal wards, with a marked increase in quarter one of 2020 (Figure 1b), almost a third of which were *K. pneumoniae* BSI.

### 3.2. Demographics of Neonates with Bloodstream Infection

Most BSI episodes affected preterm neonates (661/740; 89.3%), two-thirds of whom were born at less than 32 weeks gestation (Table 1). Overall, 640/740 (86.5%) were of low birth weight (<2500 g). The median age at HA-BSI onset was day 11 of life (IQR 7–24). The crude mortality rate for neonatal BSI (244/842) was 29.0% (95th CI 26.0–32.1). Two-thirds of deaths were directly attributable to the BSI episode (164/244; 67.2%). Compared to neonates with Gram-positive BSIs (36/231, 15.6%), neonates with Gram-negative BSIs had almost 3-fold higher odds of mortality (164/472, 34.7%; OR 2.88 [1.93–4.32]; *p* < 0.0001). Neonates with fungal BSIs showed no difference in the odds of death (9/36, 25.0%; OR 1.60 [0.73–3.48]; *p* = 0.238) compared to neonates with Gram-positive BSI episodes, although this may have been affected by the small number of fungal BSI episodes. The median length of hospital stay in BSI survivors was 44 (IQR 26–63) days.

### 3.3. Pathogen Spectrum and Antimicrobial Susceptibility of EO-BSIs

**Figure 2:**
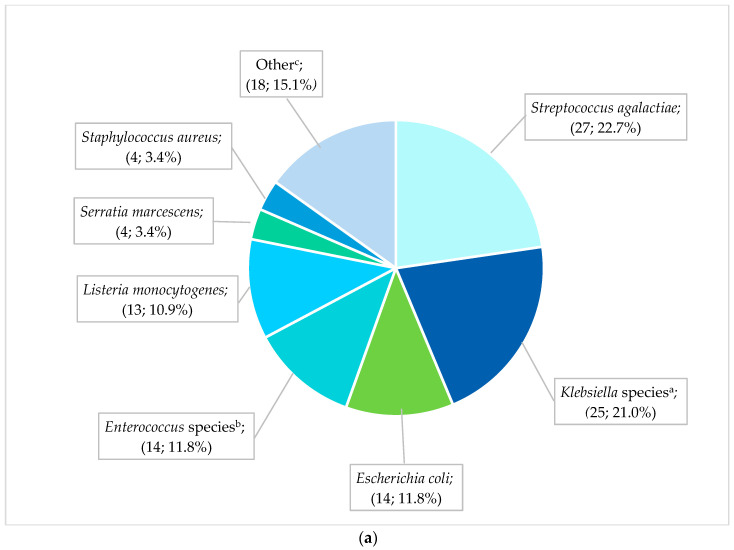

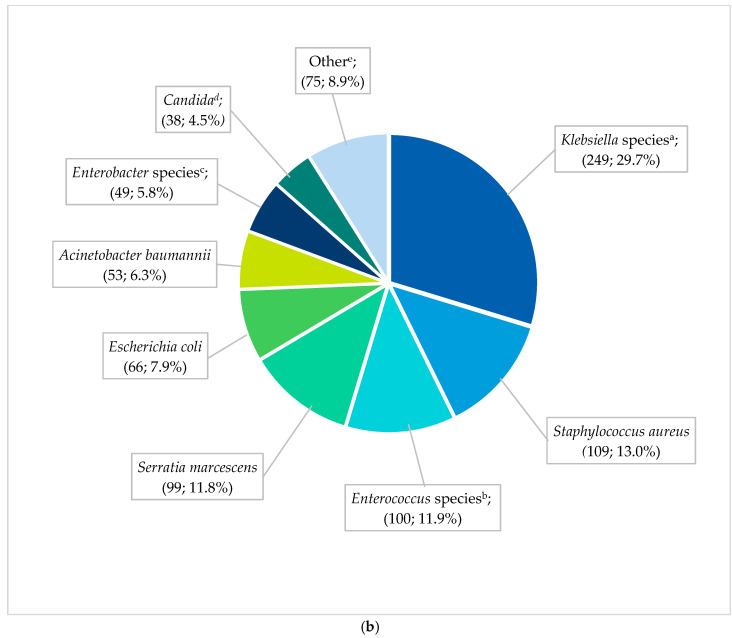
(**a**) Spectrum of EO-BSI pathogens (*n* = 119 pathogens). *BSI, bloodstream infection*. ^a^ *Klebsiella* species include *Klebsiella pneumoniae* (24) and *oxytoca* (1). ^b^ *Enterococcus* species include *Enterococcus faecalis* (12) and *Enterococcus faecium* (2). ^c^ Other includes *Acinetobacter baumannii* (3), *Pseudomonas aeruginosa* (3), *Candida pelliculosa* (2), *Enterobacter cloacae* (2), *Haemophilus influenzae* (2), *Candida albicans* (1), *Citrobacter* species (1), *Elizabethkingia meningoseptica* (1), *Serratia fonticola* (1), *Sphingomonas paucimobilis* (1), and *Stenotrophomonas maltophilia* (1). (**b**) Spectrum of neonatal healthcare-associated BSI pathogens (*n* = 838 pathogens). BSI, bloodstream infection. ^a^ *Klebsiella* species include *Klebsiella pneumoniae* (236) and *oxytoca* (13). ^b^ *Enterococcus* species include *Enterococcus faecalis* (58), *E. faecium* (40), and Enterococcus species (2). ^c^ *Enterobacter* species includes *Enterobacter cloacae* (42), Enterobacter species (3), *E. asburiae* (2), and *E. aerogenes* (2). ^d^ Candida includes *Candida albicans* (20), *C. parapsilosis* (11), *C. pelliculosa* (2), *C. lusitaniae* (1), *C. fabianii* (1), *C. krusei* (1), and *C. tropicalis* (1), Yeast: *Wickerhamomyces anomalus* (1). ^e^ Other includes *Streptococcus agalactiae* (24), *Pseudomonas aeruginosa* (18), *Stenotrophomonas maltophilia* (8), *Proteus mirabilis* (5), *Citrobacter freundii* (4), *Raoultella planticola* (4), *Burkholderia cepacia* (2), *Coagulase negative staphylococcus* (2), *Morganella morgagnii* (2), *Citrobacter sedlakii* (1), *Raoultella ornithinolytica* (1), *Staphylococcus epidermidis* (1), *Staphylococcus haemolyticus* (1), *Staphylococcus lentus* (1), and *Sphingomonas paucimobilis* (1).

The most frequent pathogens (*n* = 119) cultured from neonates with EO-BSIs were *S. agalactiae* (27; 22.7%), Klebsiella species (25; 21.0%), and *E. coli* (14; 11.8%) (Figure 2a). All cases of *S. agalactiae* and *Listeria monocytogenes* were sensitive to the first-line antibiotic regimen of ampicillin plus gentamicin. In contrast, Gram-negative pathogens exhibited substantial antibiotic resistance: 70.8% (17/24) of EO-BSI *K. pneumoniae* isolates were extended-spectrum β-lactamase (ESBL) producers, and 12.5% (3/24) were carbapenem-resistant. *K. pneumoniae* infections occurred predominantly in extremely low-birth-weight neonates (41.7% [10/24] <1 kg, and 66.7% [16/24] with a gestational age of less than 32 weeks). Most *K. pneumoniae* EO-BSIs occurred on day 2 or 3 of life (17/24; 70.8%).

### 3.4. Pathogen Spectrum and Antimicrobial Susceptibility of HA-BSIs

**Figure 3:**
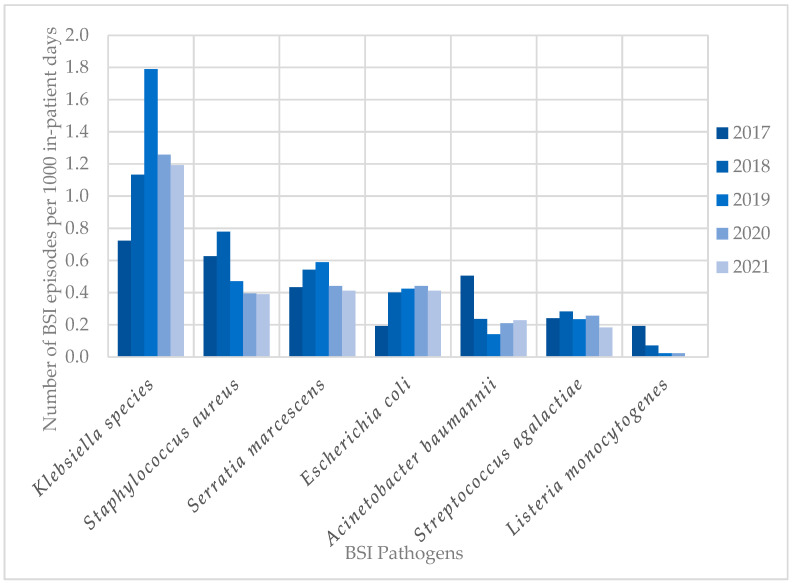
Trends in neonatal BSI pathogen incidence (2017–2021). BSI, bloodstream infection.

The most frequent pathogens (*n* = 838) cultured in HA-BSI episodes were Klebsiella species (249; 29.7%), *Staphylococcus aureus* (109; 13.0%), Enterococcus species (100; 11.9%), and *Serratia marcescens* (99; 11.8%) (Figure 2b). HA-BSI pathogens exhibited high rates of AMR, including *K. pneumoniae* (152/236 [64.4%] ESBL; 29/236 [12.3%] carbapenem-resistant), *S. aureus* isolates (66/109 [60.6%] methicillin-resistant), and *A. baumannii* (38/53 [71.7%] carbapenem-resistant). Overall, Klebsiella species remained the most frequently cultured organisms, peaking in 2019 with 1.8 BSI episodes per 1000 in-patient days (Figure 3). This coincided with the first neonatal unit CRE outbreak at the institution [13]. *S. aureus* infections steadily declined from 2018 to 2021, with similarly declining rates of methicillin resistance (21/25 [84%] in 2017 to 3/15 [20%] in 2021). Fungi were cultured in 38 HA-BSI episodes, with most being *Candida albicans* (20/38, 52.6%) or *Candida parapsilosis* (11/38, 28.9%).

### 3.5. Empiric Antibiotic Prescriptions and Antibiotic Concordance

EO-BSI episodes were treated with ampicillin/penicillin G plus gentamicin or a third-generation cephalosporin in most cases (84/113, 74.3%). Piperacillin-tazobactam plus amikacin, or a carbapenem-containing regimen (meropenem alone or with another antibiotic) was prescribed in 25/113 (22.1%) EO-BSI cases, most of which occurred on day 2 or 3 of life. HA-BSIs were treated with a carbapenem-containing regimen in 68.2% of cases (497/729) and with piperacillin-tazobactam plus amikacin in 25.7% (187/729). Colistin was included in 4.7% of HA-BSI regimens (34/729) (Table 1).

**Figure 4:**
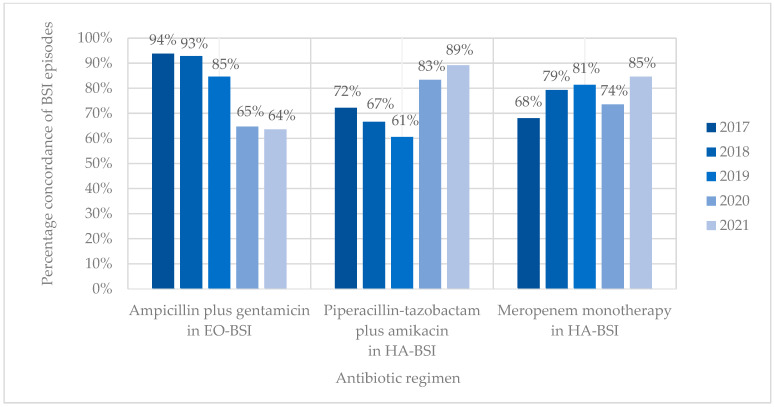
Concordance of BSI episodes with different empiric antibiotic regimens. EO-BSI, early onset bloodstream infection; HA-BSI, hospital-associated bloodstream infection.

Antibiotic coverage rates (concordance) for ampicillin plus gentamicin and EO-BSI pathogens declined from 93.8% in 2017 to 63.6% in 2021 (15/16 vs. 14/22; *p* = 0.053) (Figure 4). Antibiotic coverage rates (concordance) for HA-BSI pathogens improved from 72.2% to 89.2% for piperacillin-tazobactam plus amikacin and from 68.1% to 84.6% for meropenem between 2017 and 2021. Discordance (bug–drug mismatch) of empiric piperacillin-tazobactam plus amikacin was mostly owing to BSIs with methicillin-resistant *Staphylococcus aureus* (19/46, 41.3%) and antibiotic-resistant *K. pneumoniae* (carbapenem-resistant or ESBL-producing) (13/46, 28.3%), while discordance of empiric meropenem occurred due to CRAB (16/60, 26.7%), *E. faecium* (15/60, 25.0%), and MRSA (14/60, 23.3%) infections. Neonates with BSI who received discordant empiric antibiotic therapy were 1.5-fold more likely to die than those who received concordant antibiotic therapy (71/197 [36.0%] vs. 172/644 [26.7%]; OR 1.55 [1.10–2.17]; *p* = 0.012).

## 4. Discussion

We describe the development and implementation of the first prospective neonatal BSI surveillance system in South Africa. The data collected provides a comprehensive view of the demographic profile of neonates with BSIs, the BSI pathogen spectrum, and AMR contributing to neonatal sepsis in our setting. We documented empiric antibiotic prescriptions and assessed the appropriateness of the locally recommended empiric antibiotic regimens for the treatment of EO-BSI and HA-BSI.

This prospective neonatal BSI surveillance system was developed in response to sustained high BSI rates and frequent infection outbreaks experienced in the second largest public sector neonatal unit in South Africa [11]. A key constraint was the lack of human and financial resources to conduct continuous prospective BSI surveillance, as implemented in HIC settings. This challenge was overcome by combining the surveillance activities with the IPC nurse practitioner’s daily activities during her maternity, neonatal, and paediatric service IPC duties. The surveillance methods were simple, including a daily review of positive neonatal blood cultures at the onsite laboratory. This was followed by neonatal ward visits to advise on IPC precautions and collect demographic information and record empiric antibiotic treatment and patient outcomes. Additional antibiotic susceptibility data was obtained retrospectively from the laboratory. Neonatal BSI surveillance data were disseminated weekly via email to inform clinicians and ward managers.

Most neonates with BSIs were preterm and/or of low birth weight, in keeping with neonatal in-patient BSI data globally [8,16,20]. Most BSI episodes in this institution were healthcare-associated and caused by Gram-negative and antimicrobial-resistant pathogens. The BSI incidence density was up to seven-fold higher in the NICU compared to general neonatal wards, in keeping with the increased vulnerability to infection among critically ill neonates, frequently in need of surgical procedures and indwelling invasive devices in NICU settings. Neonates with BSI episodes had substantial mortality rates (29%) and demised rapidly following BSI onset (two-thirds of deaths were BSI-attributable).

EO-BSI is traditionally thought to reflect vertical (mother–neonate) pathogen transmission via the placenta or the genital tract. In this surveillance period, only 1 in 8 episodes were EO-BSIs. Our EO-BSI incidence rate of 2.9 per 1000 live births is comparable to the USA and Australia (reporting rates from 1.5 to 3.5 per 1000 live births) [21] but higher than that reported (0.7 per 1000 live births) by the neonIN surveillance in the United Kingdom [22]. A substantial peak was noted in quarter 2 of 2021 (8.3 per 1000 live births), which coincided with a decrease in HA-BSI infections in the same time period. It is possible that these infections represented a cluster of HA-BSIs rather than EO-BSIs, as emerging evidence suggests that HA-BSI transmission may occur earlier than 72 h of birth [6]. Rapid horizontal acquisition, rather than vertical transmission from the mother, may similarly be reflected by the finding that EO-BSI episodes caused by *K. pneumoniae* occurred late (day 2 or 3) during the “early onset” period in comparison to typical EO-BSI pathogens such as *E. coli* and *S. agalactiae*. *S. agalactiae*, *K. pneumoniae*, and *E. coli* were leading EO-BSI pathogens at our hospital, following pathogen profiles reported in a 2021 Cochrane review of EO-BSIs in LMIC [21]. *S. aureus*, although reported to be a leading cause of EO-BSI in Africa [16], was not a common isolate in our setting during this surveillance period.

HA-BSI accounted for 7 in 8 BSI episodes at our institution, with an incidence rate of 3.4 per 1000 in-patient days, which was markedly lower than that reported from a Botswanan neonatal unit (12.1 per 1000 days) [14]. The HA-BSI rate specifically in our NICU was 11.8 per 1000 in-patient days, which was higher in comparison to German NICU surveillance data (7.3 per 1000 days) [8]. The variable neonatal HA-BSI rates reported globally reflect different population risks for infection (NICU and surgical neonates, extremely low-birth-weight infants) and different surveillance definitions used. We found marked differences in the HA-BSI rates in our NICU versus general wards, likely owing to higher rates of central lines, surgical interventions, and the severity of illness in NICU patients. Almost two-thirds of HA-BSI episodes were caused by Gram-negative infections, including *K. pneumoniae*, *S. marcescens*, and *A. baumannii*, similar to the pathogen profiles described in the multi-national NeoOBS study [23].

Carbapenem resistance in *K. pneumoniae* isolates rose dramatically in 2019, reflecting a large CRE outbreak at the institution [13]. A corresponding increase in colistin use was noted, illustrating a change in prescribing behaviour in response to surveillance data. A similar event occurred in 2017, associated with an outbreak of carbapenem-resistant *A. baumannii* [23]. There was a declining number of *S. aureus* infections between 2018 and 2021, and a progressive reduction in MRSA rates over time, which has also been reported in a 20-year review of *S. aureus* neonatal sepsis in Australia [24]. Although the direct cause of the declining MRSA rates is unknown, improved hand hygiene practices and vascular device care [24] may have contributed in both settings.

Our findings on the pathogen and AMR profile of neonatal BSIs challenge the continued usefulness of the traditional time-based cut-offs for classifying EO- and HA-BSI. In LMIC, Gram-negative and AMR infections are increasingly identified from soon after birth, reflecting both the increased genital tract carriage of Gram-negative pathogens and challenges with horizontal pathogen transmission from poor infection control in delivery units [6]. Maternal bacterial infection, as well as colonisation, has been shown to be associated with increased odds of a neonate developing an infection in the first week of life [25,26]. As levels of ESBL infection and colonisation have been shown to be high in pregnant women in Africa [27], the prevention of maternal colonisation and infection is an important area to address, to prevent neonatal colonisation and infection with antibiotic-resistant organisms.

World Health Organisation (WHO)-recommended empiric antibiotics for EO-BSI (ampicillin/penicillin G plus gentamicin or a third-generation cephalosporin) were prescribed in nearly three-quarters of neonates with EO-BSI cases. Approximately one-quarter were commenced on other regimens (on day 2 or 3 of life), possibly reflecting clinicians’ suspicion of a healthcare-associated and/or antibiotic-resistant pathogen. In two-thirds of these BSI episodes, an organism was cultured that was not sensitive to the WHO-recommended empiric therapy. The high number of *K. pneumoniae* BSIs and substantial proportion that were ESBL producers or carbapenem-resistant were major drivers of increasing EO-BSI antibiotic discordance over time.

Over two-thirds of HA-BSI episodes were treated with a carbapenem-containing regimen, and a quarter with piperacillin-tazobactam plus amikacin. Colistin was empirically prescribed in <5% of regimens and predominantly during the two outbreak years (CRAB in 2017, CRE in 2019). Encouragingly, concordance of piperacillin-tazobactam plus amikacin and meropenem with HA-BSI pathogens improved over time, although the reasons for this trend are unclear. Discordance of empiric piperacillin-tazobactam plus amikacin was mostly owing to BSIs with MRSA and multi-drug-resistant *K. pneumoniae,* while discordance of empiric meropenem occurred due to CRAB, *E. faecium*, and *MRSA* infections.

Overall, one-third of neonates with a BSI died, and two-thirds of these deaths were attributable to BSI. Gram-negative infections and BSIs with discordant empiric antibiotic regimens were associated with higher odds of death. A recent global cohort study confirmed a 3-fold increase in 30-day mortality following discordant empiric antimicrobial treatment of children and neonates with a BSI [28]. Choosing the correct empiric antibiotic is therefore crucial in LMIC neonatal units and could potentially contribute to enhanced BSI survival.

This study has several limitations. Due to resource limitations, data collection was performed by a single IPC nurse practitioner, which limited the amount of clinical data included. Initial data collection was paper-based and was then transferred to an electronic Excel document, which has the risk of documentation errors. Although concordance between empiric antibiotic treatment and reported antimicrobial susceptibility patterns was noted, full in vitro sensitivities were not always available and had to be inferred in a minority of cases. Numbers of EO-BSIs per year are generally low, which will challenge the accuracy of results. A crude association between discordant therapy and mortality was documented, but a full assessment of factors related to mortality rates was not performed, which may have affected the illustrated association. Despite these limitations, this neonatal BSI surveillance data represents the first prospective study from a large public hospital, adding to the limited data of this nature in Africa. In addition, this study has documented empiric prescribing behaviours and antibiotic concordance patterns. Future neonatal BSI surveillance from other African countries would be beneficial to inform the selection of empiric neonatal sepsis treatments on the continent. Ongoing surveillance is also useful to detect infection outbreaks early, identify units most in need of IPC interventions, and monitor the effectiveness of these programmes.

## 5. Conclusions

The BSI surveillance programme (2017–2021) at this large public sector neonatal service collected useful information on pathogen and AMR trends, empiric antibiotic use, and BSI pathogen concordance with recommended empiric antibiotic regimens. Gram-negative pathogens, especially *K. pneumoniae,* remain an important cause of EO- and HA-BSIs and are associated with high rates of AMR and mortality. Discordant empiric antimicrobial therapy is also associated with an increased risk of death. The declining empiric antibiotic coverage of EO-BSI requires a change in treatment guidelines to minimise discordant therapy rates. Our findings illustrate the benefits of implementing a neonatal BSI surveillance programme and the need for ongoing surveillance at this and other facilities.

## Figures and Tables

**Table 1:** Demographic profiles of neonates with bloodstream infections (2017–2021).

Patient Characteristics	Number (%)
Number of discrete patients	740
Sex (male)	388 (52.4)
Birth weight category, *n* = 740 <1000 g 1000–1499 g 1500–2499 g >2499 g	229 (30.9) 268 (36.2) 143 (19.3) 100 (13.5)
Gestational age at birth, *n* = 740 extremely preterm (<28 weeks) very preterm (28–31 weeks) moderate–late preterm (32–36 weeks) term (>37 weeks)	170 (23.0) 313 (42.3) 178 (24.1) 79 (10.7)
**Bloodstream infection episode characteristics**	**Number (%)**
Total BSI episodes	842
BSI classification, *n* = 842 early onset BSI healthcare-associated BSI	113 (13.4) 729 (86.6)
BSI rates early onset BSI healthcare-associated BSI	2.9/1000 live births 3.4/1000 in-patient days
Total BSI pathogens isolated, *n* = 957 monomicrobial BSI polymicrobial BSI ^a^	739 (87.8) 103 (12.2)
Pathogen type, *n* = 957 Gram-negative Gram-positive fungal	620 (64.8) 296 (30.9) 41 (4.3)
EO-BSI empiric antibiotic regimen, *n* = 113 ampicillin/penicillin G + gentamicin or third-generation cephalosporin ^b^ piperacillin-tazobactam + amikacin any carbapenem-containing regimen none ^c^	84 (74.3) 8 (7.1) 17 (15.0) 4 (3.5)
HA-BSI empiric antibiotic regimen, *n* = 729 ampicillin + gentamicin or third-generation cephalosporin ^d^ piperacillin-tazobactam + amikacin any carbapenem-containing regimen any colistin-containing regimen other empiric regimen (cloxacillin, vancomycin, fluconazole, etc.) none ^e^	7 (1.0) 187 (25.7) 497 (68.2) 34 (4.7) 20 (2.7) 16 (2.2)
Concordance of empiric therapy with identified pathogen/s, *n* = 842 early onset BSI healthcare-associated BSI	85/113 (75.2) 559/729 (76.7)
Length of stay by BSI outcome, median (IQR) days BSI survivors BSI deaths	44 (26–63) 12 (7–32)
BSI episode mortality crude mortality ^f^ attributable mortality ^g^	244/842 (29.0) 164/244 (67.2)
Crude mortality by pathogen type in monomicrobial BSI, *n* = 739 Gram-negative Gram-positive fungal	164/472 (34.7) 36/231 (15.6) 9/36 (25.0)

^a^ Two or more organisms cultured, ^b^ ampicillin + gentamicin (82); cefotaxime (1); penicillin G + gentamicin (1); ^c^ rapid demise (3); life-limited condition, treatment withheld (1); ^d^ ampicillin + gentamicin (5); cefotaxime (1); cefotaxime + gentamicin (1); ^e^ delayed antibiotic initiation (9); rapid demise (3); life-limited condition, treatment withheld (3); ^f^ number of deaths as a proportion of total BSI episodes; ^g^ number of deaths within 3 days of blood culture collection as a proportion of total deaths.

## Data Availability

The research dataset is available from the corresponding author on reasonable request.

## Abbreviations

The following abbreviations are used in this manuscript:

AMR	antimicrobial resistance
BSI	bloodstream infection
CRE	Carbapenem-resistant Enterobacterales
CRAB	Carbapenem-resistant *Acinetobacter baumannii*
ELBW	extremely low birth weight
EO-BSI	early onset bloodstream infection
EONS	early onset neonatal sepsis
ESBL	extended-spectrum β-lactamase
HA-BSI	healthcare-associated bloodstream infection
HAI	healthcare-associated infection
HIC	high-income country
IPC	infection prevention and control
IQR	interquartile range
LBW	low birth weight
LMIC	low- and middle-income countries
MRSA	methicillin-resistant *Staphylococcus aureus*
NHLS	National health laboratory service
NICU	neonatal intensive care unit
OR	odds ratio
TBH	Tygerberg hospital
VLBW	very low birth weight
